# Reducing Surgeon Fatigue Through Ergonomics: Importance and Benefits in Laparoscopic Surgeries

**DOI:** 10.7759/cureus.65530

**Published:** 2024-07-27

**Authors:** Harendra Kumar, Arkadeep Dhali, Jyotirmoy Biswas, Gopal Krishna Dhali

**Affiliations:** 1 General Surgery, Dow University of Health Sciences, Karachi, PAK; 2 Gastroenterology, The University of Sheffield, Sheffield, GBR; 3 Internal Medicine, Sheffield Teaching Hospitals National Health Services (NHS) Foundation Trust, Sheffield, GBR; 4 General Surgery, College of Medicine and Sagor Dutta Hospital, Kolkata, IND; 5 Gastroenterology, School of Digestive and Liver Diseases, Institute of Postgraduate Medical Education and Research, Kolkata, IND

**Keywords:** training, laparoscopy, outcome, surgical ergonomics, general surgery

## Abstract

Laparoscopy, despite enhancing surgical outcomes, presents ergonomic challenges, such as visual-motor axis dissociation and increased cognitive load, leading to inefficiency and fatigue. Ergonomics, optimizing tasks and environments to fit human capabilities, can address these issues by designing user-friendly instruments, improving surgeon positioning, and enhancing operating room setups. These interventions reduce suturing time, alleviate discomfort, and decrease musculoskeletal disorders among surgeons. Ergonomic training for surgical teams further minimizes risk factors and promotes better body mechanics. Prioritizing ergonomics in surgical environments may lead to improved patient outcomes, greater surgeon well-being, and increased job satisfaction, highlighting its critical importance in modern surgery.

## Editorial

Although the advent of laparoscopy has improved surgical results, it has presented doctors with a unique ergonomic challenge. Laparoscopy's dissociation of the visual and motor axes, loss of depth perception, absence of direct tactile input, and narrow viewing spectrum enhance the surgeon's cognitive load, leading to decreased efficiency, a greater rate of inaccuracy, and increased fatigue [1].

Ergonomics must be introduced into the operational environment to solve this problem. This may be accomplished by understanding ergonomics and applying it to the area of laparoscopy. Ergonomics is the study of optimally fitting a person to their task or making the location and surroundings more conducive to the surgeon. It is based on a systems approach that combines anatomy, physiology, psychology, and engineering [2].

Benefits of ergonomics in laparoscopy

Using ergonomics in laparoscopy may result in advantages such as decreased suturing time, alleviation of persistent pressure discomfort, and increased surgeon comfort and efficiency. These benefits may result in less sick and injury leave, increased general well-being for the surgeon, and higher work satisfaction [2,3]. To maintain the safety and well-being of surgeons, we must recognize the importance of ergonomics in laparoscopy and develop an ergonomically favorable operating room environment that addresses the particular ergonomic difficulties of laparoscopy [2,4].

Ergonomic design of surgical instruments

The design of surgical instruments is an essential aspect of ergonomics in laparoscopy. These tools should be easy to grip and operate, with little hand strain and cramping. Furthermore, the tool's design should allow precise movement and control while lowering the surgeon's effort. An ergonomic instrument design also reduces the possibility of the tool slipping or falling during surgery, which might result in significant patient harm or injury to the surgeon [5]. For example, better instrument designs can reduce the slip of clips while placing them on the cystic duct during laparoscopic cholecystectomy.

Surgeon positioning during procedures

Positioning the surgeon's body during the process is another critical aspect of ergonomics. The surgeon watches a monitor while manipulating surgical tools into the patient's body during a laparoscopic procedure. Because of the surgeon's extended static posture, this position may cause neck, back, and shoulder pain [5]. To alleviate this problem, ergonomic operating tables may change the patient's status during surgery. Adjustable armrests, seat cushions, and back supports may also alleviate postural strain and pain [1].

Ergonomic training for surgical teams

Moreover, surgical teams may be taught to recognize and reduce ergonomic risk factors to improve operating room ergonomics. Training may include proper body mechanics, correct placement of the surgeon and patient, and ergonomic instrument handling methods. This training may assist surgeons in developing ways to reduce ergonomic risk factors, such as frequent stops, changing postures, and stretching [1,4].

Preventing work-related musculoskeletal injuries

Ergonomics are critical in preventing work-related musculoskeletal injuries among surgeons. Musculoskeletal disorders (MSDs) are the most prevalent kind of occupational injury among healthcare workers, accounting for most missed workdays [3]. Ergonomic modifications may lower the critical risk factors for MSDs, allowing employees to be more efficient, productive, and satisfied at work [3-5].

Ergonomic interventions

Ergonomic interventions for deducing surgeon fatigue are elaborated in Table 1.

**Table 1:** Ergonomic interventions for reducing surgeon fatigue in laparoscopic surgeries

Intervention	Description
Adjustable Furniture	Providing furniture that can be adjusted to suit the surgeon's physical dimensions, including height-adjustable chairs, adjustable tables, and adjustable footrests.
Proper Lighting and Ventilation	Ensuring that the operating room has adequate lighting and ventilation, which can reduce fatigue and improve visibility during the surgery.
Monitor Arms	Providing adjustable monitor arms that allow the surgeon to position the monitor in a comfortable and ergonomic position.
Ergonomic Chair	Providing an ergonomic chair with lumbar support and adjustable armrests to reduce muscle strain and discomfort.
Workflow Optimization	Reducing unnecessary movements and optimizing the workflow of the surgery to reduce the physical and cognitive burden on the surgeon.
Staff Rotation	Implementing a staff rotation system that allows surgeons to take breaks and switch tasks to reduce physical and mental fatigue.
Task Simplification	Simplifying tasks and reducing unnecessary complexity to reduce the cognitive burden on the surgeon.
Training	Providing training on ergonomic principles and proper body mechanics to help surgeons work more efficiently and safely.

To alleviate physical stress on the body, ergonomic enhancements include offering adjustable chairs, monitor arms, and footrests. Proper lighting and ventilation also help relieve tiredness and increase sight during surgery. Furthermore, offering an ergonomic chair with lumbar support and armrests that adapt to the surgeon's height may help prevent muscular strain and pain [1].

Conclusion

To summarize, ergonomics is crucial for improving surgical outcomes, reducing the risk of work-related injuries, and increasing surgeon comfort and job satisfaction. To ensure a safe and productive work environment, surgeons and their colleagues should prioritize the design of ergonomic operating rooms, surgical gear, and training programs. Investing in ergonomics may result in better patient outcomes, a more efficient surgical process, and happier, healthier clinicians.
